# Optimization and validation of a cost‐effective protocol for biosurveillance of invasive alien species

**DOI:** 10.1002/ece3.7139

**Published:** 2021-02-10

**Authors:** Yoamel Milián‐García, Robert Young, Mary Madden, Erin Bullas‐Appleton, Robert H. Hanner

**Affiliations:** ^1^ Department of Integrative Biology University of Guelph Guelph ON Canada; ^2^ Canadian Food Inspection Agency Government of Canada Ottawa ON Canada

**Keywords:** 16S, biomonitoring, biosurveillance, COI, eDNA metabarcoding, invasive alien species, ITS, salt trap solution

## Abstract

Environmental DNA (eDNA) metabarcoding has revolutionized biodiversity monitoring and invasive pest biosurveillance programs. The introduction of insect pests considered invasive alien species (IAS) into a non‐native range poses a threat to native plant health. The early detection of IAS can allow for prompt actions by regulating authorities, thereby mitigating their impacts. In the present study, we optimized and validated a fast and cost‐effective eDNA metabarcoding protocol for biosurveillance of IAS and characterization of insect and microorganism diversity. Forty‐eight traps were placed, following the CFIA's annual forest insect trapping survey, at four locations in southern Ontario that are high risk for forest IAS. We collected insects and eDNA samples using Lindgren funnel traps that contained a saturated salt (NaCl) solution in the collection jar. Using cytochrome c oxidase I (COI) as a molecular marker, a modified Illumina protocol effectively identified 2,535 Barcode Index Numbers (BINs). BINs were distributed among 57 Orders and 304 Families, with the vast majority being arthropods. Two IAS (*Agrilus planipennis* and *Lymantria dispar*) are regulated by the Canadian Food Inspection Agency (CFIA) as plant health pests, are known to occur in the study area, and were identified through eDNA in collected traps. Similarly, using 16S ribosomal RNA and nuclear ribosomal internal transcribed spacer (ITS), five bacterial and three fungal genera, which contain species of regulatory concern across several Canadian jurisdictions, were recovered from all sampling locations. Our study results reaffirm the effectiveness and importance of integrating eDNA metabarcoding as part of identification protocols in biosurveillance programs.

## Graphical abstract

In the present study, we optimized and validated a fast and cost‐effective eDNA metabarcoding protocol for biosurveillance of plant IAS and characterization of insect and microorganism diversity. Forty‐eight traps were placed, following the CFIA's annual forest insect trapping survey, at four locations in southern Ontario that are high risk for forest IAS. We collected insects and eDNA samples using Lindgren funnel traps that contained a saturated salt (NaCl) solution in the collection jar. Our study results reaffirm the effectiveness and importance of integrating eDNA metabarcoding as part of identification protocols in biosurveillance programs.

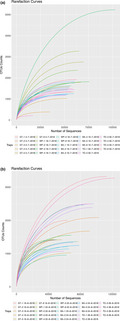

## 1 INTRODUCTION

Metabarcoding has become an effective method for molecular‐based biomonitoring programs and biosurveillance for invasive alien species (IAS; Makiola et al., 2020). This molecular technique involves high‐throughput sequencing (HTS), which facilitates the identification of multiple species using environmental DNA (eDNA) extracted from complex ecological samples (Creer et al., 2016; Taberlet et al., 2012). Incorporating HTS into biosurveillance programs is advantageous as it can provide results faster than conventional identification methods, thereby allowing for early detection of species of concern, including IAS (Ruppert et al., 2019). The ability to obtain results fast, the existence of curated DNA reference libraries (Nilsson et al., 2019; Quast et al., 2013; Ratnasingham & Hebert, 2007), and the continued drop in the cost of HTS platforms have contributed to metabarcoding's rise in popularity in environmental monitoring (Cristescu, 2014; de Kerdrel et al., 2020). Furthermore, as DNA reference libraries with associated morphological species identifications continue to grow, the time, effort, and resources that would otherwise be put toward morphological identification of specimens can be saved through the use of molecular identification methods. This is significant since the number of taxonomists available to conduct species identifications is declining. Reference libraries, therefore, provide a permanent repository of traditional taxonomic expertise that can be used with the appropriate molecular identification tools as needed (Cristescu, 2014).

The mitochondrial DNA region coding for cytochrome c oxidase I (COI) enzyme has been recognized as the primary marker for metabarcoding in the animal kingdom (Hebert et al., 2003). Similarly, the nuclear ribosomal internal transcribed spacer (ITS) and the 16S ribosomal RNA gene have been adopted as the fungal and bacterial markers, respectively (Klindworth et al., 2013; Seifert, 2009). Multiple reference databases and workbenches for data collection and analysis are currently available for these markers as well as a growing number of sequence records (e.g., BOLD has 8,099,249 records for COI as of November 20, 2020; Nilsson et al., 2019; Quast et al., 2013; Ratnasingham & Hebert, 2007, 2013, and UNITE has 2,480,043 for ITS as of November 20, 2020). However, despite the plethora of protocols available for eDNA metabarcoding and the drop in the cost of HTS over the years, there is limited information available on cost‐effectiveness (Elbrecht & Steinke, 2019; Ji et al., 2013; de Kerdrel et al., 2020). Biodiversity monitoring and biosurveillance programs would benefit from the inclusion of HTS protocols to accelerate the detection of species of concern and reduce costs associated with processing large numbers of individual specimens (Giovani et al., 2020; Piper et al., 2019). On a broader scale, cost‐effectiveness is essential given that only a fraction of the global biodiversity (including IAS and other pest species) has been described to date, and several billions of dollars may be needed to complete this enormous endeavor (Carbayo & Marques, 2011).

Pest insects have a negative impact on Canada's forests and are second only to wildfires in their effect ("The State of Canada's Forests. Annual Report 2018.", n.d.). Pest insects that are IAS are considered a byproduct of anthropogenic activities and can provoke significant economic and biodiversity losses (Westphal et al., 2008). For instance, IAS are capable of destroying about 400,000 ha of forest every year in Canada (Government of Canada, 2013). Nonmanufactured wood packaging and loose wood dunnage are high‐risk pathways for the introduction of IAS, particularly wood‐boring beetles (e.g., Cerambycidae and Buprestidae families), like the Asian long‐horned beetle (*Anoplophora glabripennis*) and the emerald ash borer beetle (*Agrilus planipennis*) (CFIA, 2017). Current pathway‐based biosurveillance programs led by the Canadian Food Inspection Agency (CFIA) includes placing traps at sites that are at a high risk for IAS, such as industrial zones receiving international commodities associated with nonmanufactured wood packaging and dunnage. The trapped insects remain in the fluid of collection jars until specimens are decanted and referred to the CFIA Entomology laboratory for morphological identification. As organisms interact with their environment, whether it be the fluid of a collection jar or a plant, they shed DNA into it (Adams et al., 2019; Tab erlet et al., 2018). The eDNA extracted from the collection fluid can then be used to identify insects considered IAS, native pests, and accompanying microorganisms using molecular methods such as eDNA metabarcoding (Pawlowski et al., 2020; Taberlet et al., 2018).

Traditionally, plant health trapping survey protocols for insect pest detection have used alcohol‐based collection fluids (e.g., ethanol or propylene glycol) to preserve biological material for morphological identification (CFIA, 2017). Recent protocols have replaced alcohol‐based fluids with saturated salt solutions due to advantages over the previous chemistries including lower cost, less storage space requirements, low toxicity to humans, fewer regulatory constraints for laboratories and inspectors, nonflammability, and lower evaporation rate (Young et al., 2020). Salt solutions have also proven to be satisfactory for preserving morphological structures of captured specimens (Young et al., 2020) and for the preservation of water samples for eDNA analysis (Williams et al., 2016). Therefore, the standardization and validation of a cost‐effective protocol for eDNA metabarcoding analysis using salt trap solutions are of crucial interest. The current study focusses on the optimization and validation of a protocol for eDNA metabarcoding of organisms captured during regulatory plant health monitoring surveys in southern Ontario, Canada. Special attention will be placed on the wood‐boring beetles as well as regulated pathogenic bacteria and fungi inadvertently collected in the traps.

## 2 MATERIALS AND METHODS

### 2.1 Target pests

These included all insect IAS found in Canada as well as pests that impact plant health and are regulated by the CFIA (30 taxa; https://www.inspection.gc.ca/plant‐health/plant‐pests‐invasive‐species/insects/eng/1307077188885/1307078272806). The surveillance protocol targeted long‐horned beetles (*A. glabripennis*, *Anoplophora chinensis*, *Tetropium fuscum*, *Tetropium castaneum*, and *Aromia bungii*) which are wood‐boring IAS (CFIA, 2017). Also, we explored 39 fungi and eight bacteria recognized as plant pests and regulated by the Canadian government (https://www.inspection.gc.ca/plant‐health/plant‐pests‐invasive‐species/regulated‐pests/eng/1363317115207/1363317187811). The eight bacterial species represent seven different genera: *Brenneria salicis*, *Clavibacter michiganensis* subsp. *sepedonicus*, *Pseudomonas syringae pv*. *cannabina*, *Ralstonia solanacearum*, *Xanthomonas campestris pv*. *cannabis*, *Xanthomonas populi*, *Xylella fastidiosa*, and *Xylophilus ampelinus*.

### 2.2 Collection locations

**FIGURE 1:**
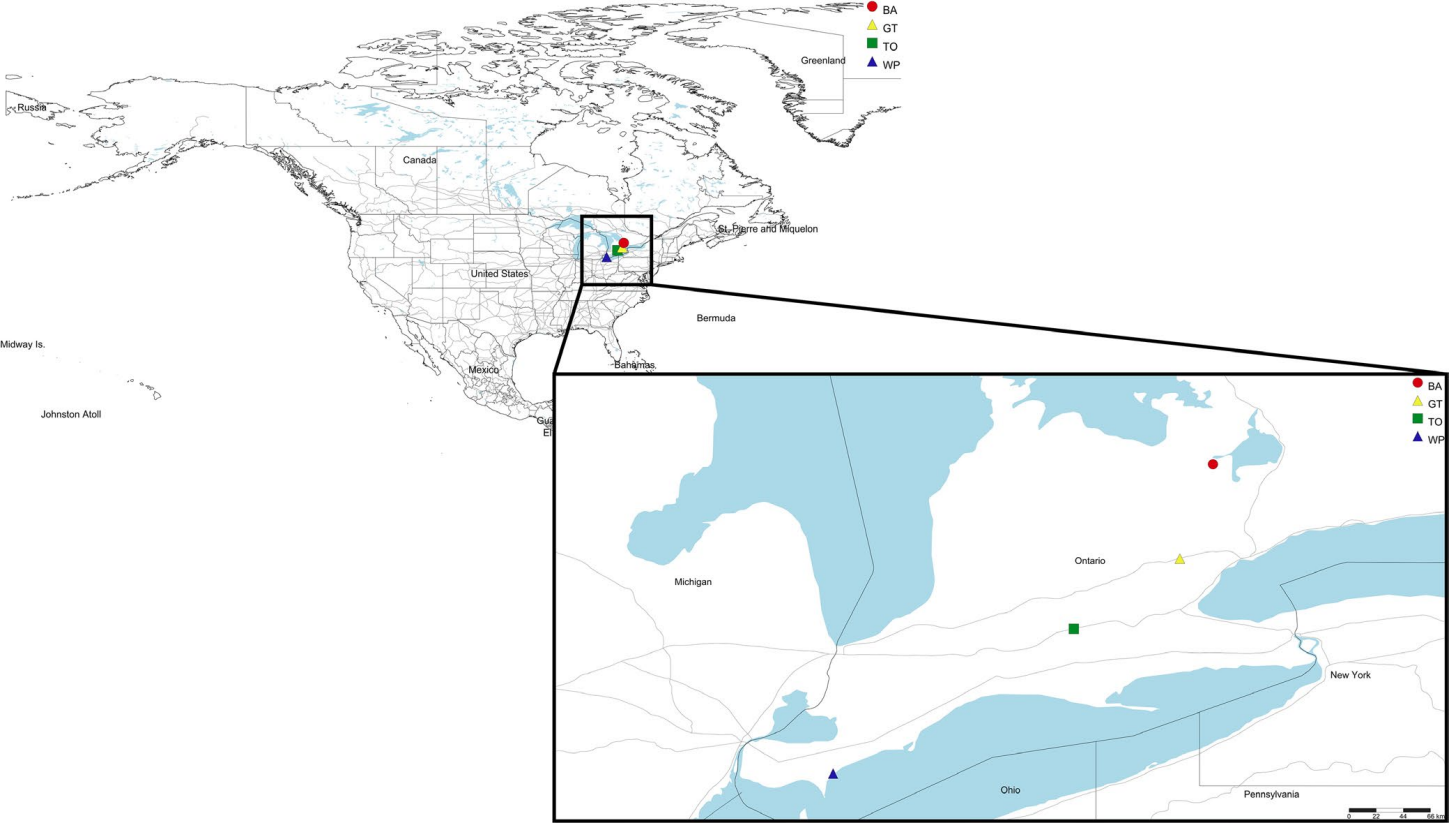
Map showing collection locations in Ontario, Canada. Inset indicates Southern Ontario. Barrie (BA), Halton Hills (GT), Woodstock (TO), and Chatham‐Kent (WP)

Lindgren funnel traps were placed at four locations in Southern Ontario, Canada, with six sample sites (traps) at each location (Figure 1). The traps represented a subset of those deployed following the CFIA's annual regulatory survey program (CFIA's Plant Health business line), which is aimed at detecting insects introduced through high‐risk pathways. Locations were selected based on susceptibility to IAS, accessibility, sufficient area to accommodate six traps spaced approximately 25–30 meters apart, and limited public access to avoid vandalism. Six traps were then distributed at these sites near species of trees known to be hosts to the target IAS and that were showing evidence of stress/decline, or damage, indicating the possible presence of IAS. One location was in Halton Hills (GT) and had traps situated near a Municipal landfill and railroad track. A second location was in a 241‐hectare park of Carolinian forest that is situated along Lake Erie in Chatham‐Kent (WP). A third location, Barrie (BA), was within a wooded area close to railway tracks that experiences a high volume of import traffic. A fourth and final location was in Woodstock (TO), a city that includes manufacturing facilities that import commodities packaged using wood materials. All four locations are less than 215 km from the US–Canada border and could presumably be exposed to traffic of imported wood materials carrying IAS and other pests.

### 2.3 Collection protocol

Lindgren funnel traps were equipped with Ultra High Release Ethanol (UHR) Lures (Synergy Semiochemical Corp), a type of lure that targets all longhorn beetles, and a collection cup that contained approximately 100 ml of a table salt‐saturated solution (2 kg of sodium chloride per 5 L of water). Traps were placed at sampling sites during the summer following CFIA's Survey Protocol: Invasive Alien Species Forestry Trapping (2017). The first set of traps was collected in July 2018 and the second in August 2018, approximately 4 weeks after having been placed. With four locations, six sample sites, and two collection periods, a total of 48 field samples were collected for the study. All specimens captured in the traps were decanted from the solution and sent to the CFIA's entomology Laboratory. Salt trap solutions were transported to the Hanner Laboratory at the University of Guelph, Ontario, Canada, for temporary storage at −80℃ followed by processing and analysis.

### 2.4 Sample filtration

Salt solutions were filtered through a nitrocellulose mixed ester membrane filter (pore size 1 µm, diameter 47 mm, Sterlitech). The filter was mounted onto a magnetic filtration cup (Pall) and secured to a 3‐piece manifold connected to an GAST vacuum pump (GAST Manufactured, Inc). All supplies were sterilized with 50% bleach or ELIMINase (Decon Labs) before filtration.

The amount of debris within the salt solutions varied greatly across samples and would sometimes cause clogging of membrane pores. Therefore, most samples required multiple membranes to filter the entire volume, resulting in a total of 110 membranes for the 48 samples. The membranes were stored at −80°C until DNA extraction could be completed. As a negative control, one sample of saturated table salt solution prepared in the laboratory was also filtered. All membranes were stored in new Ziploc bags at −80°C until processing for eDNA extraction.

### 2.5 eDNA extraction

We used a modified CTAB buffer (Coyne et al., 2005; Dempster et al., 1999) (2% w/v cetyltrimethylammonium bromide, 2% w/v polyvinylpyrrolidone, 1.4M NaCl, 100 mM Tris‐HCl, 20 nM EDTA). This buffer was used due to reported success in retrieving good quality eDNA yield (Barnes et al., 2014; Dougherty et al., 2016; Renshaw et al., 2015; Turner et al., 2014).

The eDNA extraction steps described below were performed on the 110 membranes and the negative control. To extract the eDNA, we used the Dougherty et al. (2016) protocol with some minor modifications. Each filter paper was allowed to thaw and then cut into quarters using new razor blades. Each quarter was placed into individual 2‐ml microcentrifuge tubes containing ~250 mg of 1‐mm‐diameter glass beads, and 500 μl of CTAB buffer prewarmed to 65°C in a heat block. The filter paper quarters were pulped using the TissueLyser II (Qiagen) at a frequency of 30 Hz for one minute and then incubated at 65°C for one hour in a heat block. Following incubation, each tube received 500 μl of 24:1 chloroform–isoamyl alcohol and was briefly vortexed. The aqueous phase containing the eDNA was separated from the chloroform phase by centrifuging the tubes at 13,000 *g* for 15 min to enable phase separation. After two passes with 24:1 chloroform–isoamyl alcohol, the aqueous phase (approximately 500 μl) was transferred to new 2.0‐ml Eppendorf tubes and mixed with 500 μl of isopropanol, and 200 μl of a 5 M NaCl solution. Tubes were briefly vortex and stored at −20°C overnight to facilitate precipitation of eDNA. Tubes were then centrifugated at 13,000 *g* for another 15 min to pellet the eDNA. The supernatant was removed by pipetting, and 200 μl of 70% ethanol was added to wash the pellet. After centrifugation at 13,000 *g* for 15 min, the ethanol was removed by pipetting and replaced anew. The contents were again briefly vortexed and centrifuged at 13,000 *g* for 15 min. The ethanol was removed for a final time, and the tubes were then placed in a fume hood to allow any remaining ethanol to evaporate at room temperature (~1 hr). Once the eDNA pellet was dry, it was then resuspended in 25 μl 1X TE buffer (prewarmed at 70°C) to favor DNA dilution. eDNA extracts from each quarter belonging to the same trap were pooled. A 5 μl subsample was used to visually assess the presence and quality of eDNA on a 1% agarose gel electrophoresis. The concentration of eDNA (ng/μl) was determined by fluorometry (Qubit). All eDNA extracts with concentrations over 5 ng/μl were diluted to 5 ng/μl with 10 mM Tris pH = 8.5 This was done to normalize the amount of starting material per sample and to reduce the effect of potential PCR inhibitors in the samples. Eight samples exhibited signals of inhibition by showing no amplification during the first PCR of library preparation (see below). These samples were treated to remove inhibitors using a 1x NGS magnetic beads (Macherey‐Nagel) ratio, following manufacturer's protocols. Following quality control steps, the remaining volume of solution containing eDNA extract was placed in 1.5‐ml LoBind Eppendorf tubes and stored at −20°C.

### 2.6 Library preparation

For COI and ITS library preparation, we adapted the “16S Metagenomic Sequencing Library Preparation Protocol” (Illumina) while no modifications were needed for 16S (“16S Metagenomic Sequencing Library Preparation”, 2013).

#### 2.6.1 First PCR

The first PCR was conducted in 25 μl reaction volumes on an Eppendorf Mastercycler thermal cycler with each reaction containing 2.5 μl of eDNA template, 12.5 μl of 2X KAPA HiFi HotStart Ready Mix (Roche Diagnostics), and 0.2 μM of primers with Illumina adaptors (Table 1). The eDNA target fragment lengths to amplify were ~407 base pairs (bp) for COI, 460 bp for 16S, and 500–600 bp for ITS. Cycling conditions were as follows: COI: 94°C (120 s) followed by five cycles at 94°C (40 s), 45°C (40 s), 72°C (60 s); then 35 cycles of 94°C (40 s), 51°C (40 s), 72°C (60 s); and a final extension of 72°C (5 min) (Braukmann et al., 2019); 16S: 95°C (180 s), 25 cycles of 95°C (30 s), 55°C (30 s), 72°C (30 s), and a final extension of 72°C (300 s) (“16S Metagenomic Sequencing Library Preparation”, 2013); ITS: 95°C (120 s), 40 cycles at 95°C (30 s), 55°C (30 s), 72°C (60 s), and a final extension of 72°C (600 s) (Beeck et al., 2014). A negative control was included for every batch of samples amplified. All PCR products were visualized on 1% agarose gel to check for proper amplification and fragment size of the amplicons. PCR products were purified using a 0.8x NGS magnetic beads (Macherey‐Nagel) ratio following the manufacturer's protocols.

**TABLE 1:** Illumina adapters and primers (sequence 5′‐3′) from the literature used in HTS for COI, 16S, and ITS

Adapter overhang sequences		
Forward overhang	5′ TCGTCGGCAGCGTCAGATGTGTATAAGAGACAG‐[locus‐specific sequence]	“16S Metagenomic Sequencing Library Preparation” (2013)
Reverse overhang	5′ GTCTCGTGGGCTCGGAGATGTGTATAAGAGACAG‐[locus‐specific sequence]	
COI Primers		
MLepF1	GCTTTCCCACGAATAAATAATA	Hajibabaei et al. (2006)
RonMWASPdeg	GGWTCWCCWGATATAKCWTTTCC	M. A. Smith (unpublished); Hernández‐Triana et al. (2014)
LepR1	TAAACTTCTGGATGTCCAAAAAATCA	Hebert et al. (2004); Hernández‐Triana et al. (2014)
HCO2198	TAAACTTCAGGGTGACCAAAAAATCA	Folmer et al. (1994)
16S Primers		
16S Forward	CCTACGGGNGGCWGCAG	“16S Metagenomic Sequencing Library Preparation” (2013)
16S Reverse	GACTACHVGGGTATCTAATCC	
ITS Primers		
ITS86_F	GTGAATCATCGAATCTTTGAA	Beeck et al. (2014)
ITS4_R	TCCTCCGCTTATTGATATGC	

Note

COI primers have been used as cocktails, combining 1 to 1 volume of the two forwards (forward cocktail) and the two reverse (reverse cocktail) primers. References are also shown.

#### 2.6.2 Second PCR

The purified products resulting from the first PCR were used as the template for the second PCR. As per standard methods for eDNA library preparation, the second PCR was conducted in a separate room and using different PCR workstations than the first PCR. The second PCR used unique index primer combinations for each sample. The sequences for the index primers were equivalent to the Nextera XT Index Kit (Illumina), but synthesized de novo using the services from Integrated DNA Technologies (IDT) and prepared at Advanced Analysis Centre (AAC) at the University of Guelph. This PCR was conducted in 50 μl reaction volumes on an Eppendorf Mastercycler using a unique index primer combination for each sample. Each reaction contained 5 μl of previously cleaned PCR product, 5 μl of each index primer (10 μM), 25 μl of 2X KAPA HiFi HotStart Ready Mix (Roche Diagnostics), and 10 μl of molecular biology grade water. Cycling conditions were as follows: 95°C (180 s), eight cycles at 95°C (30 s), 55°C (40 s), 72°C (30 s), and a final extension at 72°C (300 s) (“16S Metagenomic Sequencing Library Preparation”, 2013). PCR products were visualized on 1% agarose gel first and purified using a 0.6x NGS magnetic beads ratio (Macherey‐Nagel), following manufacturer's protocols.

### 2.7 High‐throughput sequencing

Sequencing of COI, 16S, and ITS marker regions was performed at the Genomics Facility of AAC at the University of Guelph. For quality control purposes, each sequence library was first normalized using SequalPrep Normalization Kit (Thermo Fisher Scientific), pooled, and quantified with the Qubit dsDNA High Sensitivity assay kit (Thermo Fisher Scientific), and finally checked for fragment size in a Bioanalyzer High Sensitivity DNA Chip (Agilent). After passing quality control, libraries were sequenced on an Illumina MiSeq System using a MiSeq reagent kit, version 3 (600 cycles). Each sample was analyzed based on retaining 1% of the total capacity of the run. Sequencing reads were demultiplexed, and the adapters trimmed with the MiSeq Reporter software generating two paired‐end FASTQ raw data files.

### 2.8 Cost analysis

We conducted a simple cost analysis that compared the cost of processing one sample (from eDNA extraction to MiSeq sequencing) based on the modified protocol described here versus the original “16S Metagenomic Sequencing Library Preparation Protocol” (Illumina) (Table  2).

**TABLE 2:** Approximate cost analysis for processing one sample, including filtering, eDNA extraction, library preparation, and MiSeq sequencing at the Genomics Facility of the Advanced Analysis Centre (AAC) at the University of Guelph

Laboratory steps for processing one sample	Cost per step CAD/USD^b^ (this study)	Cost per step CAD/USD^b^ (Illumina protocol)
Filters (pore size 1 μm, diameter 47 mm; an average of 2.5 per trap)	2.5	2.5
CTAB extraction method (4 per filter)	2.5	2.5
DNA quantification (Qubit) and normalization	2.5	2.5
First PCR (pooled extracts per filter)	5	5
Magnetic beads (at all stages)	0.7	7
Second PCR (Indexing reaction and primers^a^)	7	25
HTS Sequencing (Illumina MiSeq)	30	30
Overall	50.2 CAD/36.7 USD	74.5 CAD/54.5 USD

Note

Processing steps are compared to library preparation costs of Illumina's 16S Metagenomic Sequencing Library Preparation (“16S Metagenomic Sequencing Library Preparation”, 2013). Shaded cells represent steps common to both protocols where modifications were completed to reduce the protocol's total cost.

Nonhighlighted fields represent steps common to both protocols where no modifications were needed. The costs apply to all three molecular markers: COI, 16S, and ITS.

^a^ Illumina protocol adapted by the AAC at the University of Guelph.

^b^ Currency exchange rates (CAD/USD) as of 09 August 2020:1 CAD = 0.73105.

### 2.9 Data analysis

Quality control of the FASTQ raw data files was explored using FastQC as per base sequence quality scores, per base sequence content, sequence length distribution, sequence duplication levels, overrepresented sequences, and adapter content. (http://www.bioinformatics.babraham.ac.uk/projects/fastqc/).

#### 2.9.1 COI

HTS data visualization, validation, analysis, and storage were conducted in the multiuser platform Multiplex Barcode Research And Visualization Environment (mBRAVE; http://mbrave.net/). The parameters in the online platform were set as follow: (a) trimming: trim front: 30 bp; trim end: 30 bp; trim length 450 bp; (b) filtering: min QV: 20 qv; min length: 350 bp; max bases with low QV [˂20]: 25%; max bases with ultralow QV [˂10]: 5%; (c) preclustering threshold: none; ID distance threshold: 3%; minimum OTU size: 5; operational taxonomic unit (OTU) threshold: 2%; (d) assembler min overlap: 20 bp, assembler max subs: 5 bp. An mBRAVE algorithm for removing chimeras was also applied to the data; (e) queries against Barcode of Life (BOLD) libraries in the following order: 1.—SYS‐MBRAVEC: System Reference Library—Standard Contaminants Based on Reagent Production; 2.—SYS‐HUMC: System Reference Library—Human Contamination Check; 3.—SYS‐CRLBACTERIA: System Reference Library for mBRAVE ID Engine—Bacteria COI; 4.—SYS‐CRLPROTISTA: System Reference Library for mBRAVE ID Engine—Protista COI; 5.—SYS‐CRLCHORDATA: System Reference Library for mBRAVE ID Engine—Chordata; 6.—SYS‐CRLAVES: System Reference Library for mBRAVE ID Engine—Aves; 7.—DS‐PLANTP20: CFIA‐Plant pests‐invasive species (insects) update February 2020; 8.—DS‐VBEETLES: Vectors of *Bretziella fagacearum* update February 2020; 9.—DS‐CUL2020: *Culicoides* Database Update: February 2020; 10.—SYS‐CRLINSECTA: System Reference Library for mBRAVE ID Engine—Insecta; 11.—SYS‐CRLNONINSECTARTH: System Reference Library for mBRAVE ID Engine—Non‐Insect Arthropoda; and 12.—SYS‐CRLNONARTHINVERT: System Reference Library for mBRAVE ID Engine—Non‐Arthropoda Invertebrates. Sequences were matched to BINs in mBRAVE. Essentially, a BIN (Barcode Index Number) is an alphanumeric code that corresponds to a tight cluster of closely related species haplotypes. BINs are a good proxy for actual biological species (Ratnasingham & Hebert, 2013).

A second pipeline for data analysis was conducted to validate the identity of the IAS recognized by mBRAVE. COI FASTQ raw data files were analyzed sequentially in Geneious Prime, version 2020.1.1 (Biomatters, Ltd.) as follows: (a) Paired reads were set; (b) sequences were trimmed with BBDuk version 37.25, keeping a minimum sequence quality of 20 Phred (i.e., 99% base call accuracy), and a minimum length of 200 bp; (c) paired‐end reads were merged with BBMerge version 37.25; (d) duplicates were removed with Dedupe version 37.25; (e) clustering was completed by de novo assembly using Geneious assembler, as well as minimum overlap identity of 98%; and (f) The Basic Local Alignment Search Tool (BLAST) was used to compare our sequence library against the National Center for Biotechnology Information (NCBI) database. Rarefaction curves expressed as the number of operational taxonomic units (OTU) identified per trap versus the number of sequences related to the OTUs identified were built in R version 3.6.1 (R Core Team, 2019).

#### 2.9.2 16S

16S molecular marker data were analyzed sequentially using Geneious Prime, version 2020.1.1 (Biomatters, Ltd.) as follows: (a) Paired reads were set; (b) sequences were trimmed with BBDuk version 37.25, keeping a minimum sequence quality of 20 Phred (i.e., 99% base call accuracy), and a minimum length of 200 bp; (c) paired‐end reads were merged with BBMerge version 37.25; (d) duplicate sequences were removed with Dedupe version 37.25; and (e) exact sequence variants were classified using the 16S Biodiversity tool implemented in Geneious Prime, version 2020.1.1 (Biomatters, Ltd.). This tool runs the Ribosomal Database Project (RDP) Classifier, version 2.12, which assigns a taxonomy to the genus level (Wang et al., 2007) and includes a bootstrap confidence estimate for each sequence by comparing them to a bacterial and archaeal 16S rRNA database (Wang et al., 2007). Search results were queried against the list of bacterial species regulated by Canada. Graphs of bacterial diversity were produced using the Krona metagenomic visualization tool (Ondov et al., 2011).

#### 2.9.3 ITS

Similar to 16S, data analysis of ITS was carried out in Geneious Prime, version 2020.1.1 (Biomatters, Ltd.) as follows: (a) Paired reads were set; (b) sequences were trimmed with BBDuk version 37.25, keeping a minimum sequence quality of 20 Phred (i.e., 99% base call accuracy), and a minimum length of 200 bp; (c) paired‐end reads were merged with BBMerge version 37.25; (d) duplicates were removed with Dedupe version 37.25; (e) chimera reads were removed using UNITE as a reference library; (f) clustering was done by de novo assembly using Geneious assembler and custom sensitivity, as well as minimum overlap identity of 98%; and (g) The Basic Local Alignment Search Tool (BLAST) was used to compare our sequence library against the Internal Transcribed Spacer database of the National Center for Biotechnology Information (NCBI). Search results were ordered by E‐value (in ascending order) and then filtered by sequence length (≥200 bp) and percentage of pairwise identity (≥98%). The filtered list was queried against the list of fungal species regulated by Canada. Graphs of fungal diversity were generated for the most species‐rich genera (≥10 species) using R (R Core Team, 2019).

## 3 RESULTS

### 3.1 COI

**FIGURE 2:**
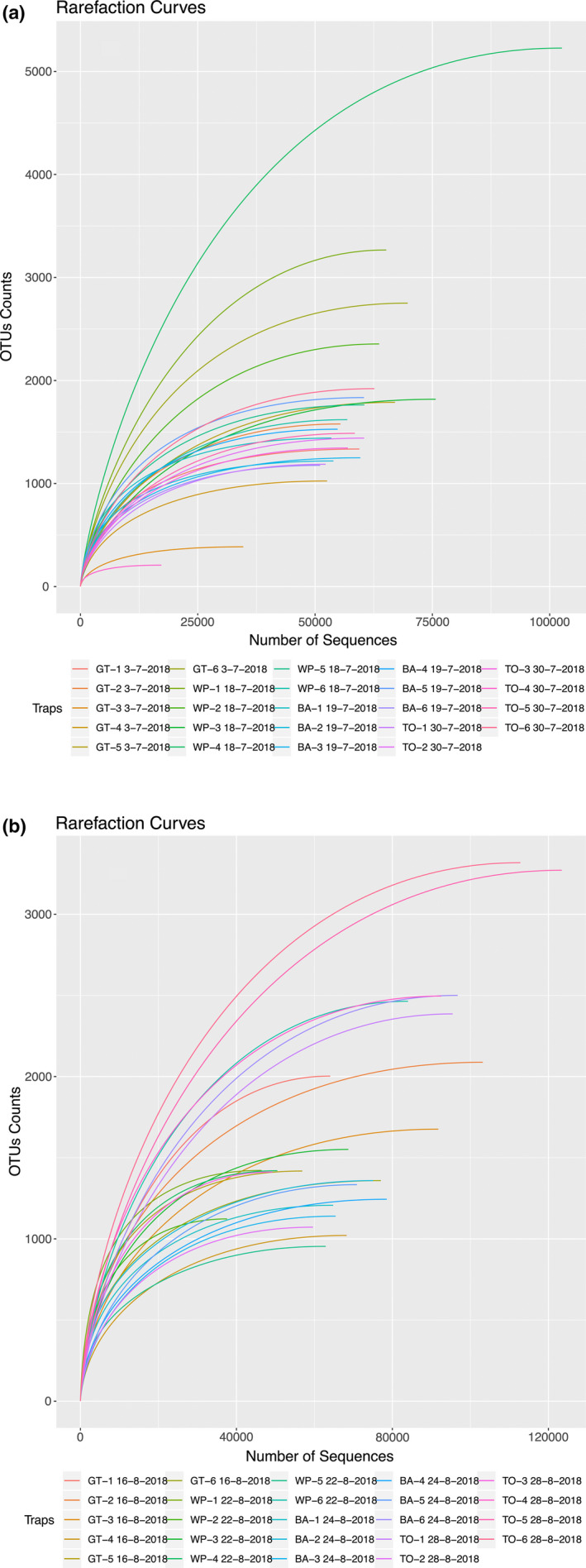
Rarefaction curves representing the number of operational taxonomic units (OTUs) versus the number of corresponding sequences for each sample site in July 2018 (a) and August 2018 (b)

All samples amplified successfully with the primer combination chosen. Fragment sizes were at the expected length of approximately 407 nucleotides for all PCR steps during library preparation. The Illumina MiSeq run generated a total of 9,034,668 reads for the 48 traps analyzed in this study with the number of reads per sample ranging from 102,571 to 338,642 (Table 3). The percentage of filtered reads was lower than 25% for all the traps except for two (WP‐1‐18‐7‐18_COI, 60.43% and BA‐6_19‐7‐18_COI, 25.73%) (Table 3). The number of suspected species present through the identification of unique BINs per trap varied from 60 to 313. The lowest number of BINs was attributed to traps placed at the Barrie (BA) location (BA‐1_24‐8‐18_COI, BA‐3_24‐8‐18_COI, and BA‐6_24‐8‐18_COI collected on August 2018) (Table 3). The highest number of BINs was recovered in traps placed at the Woodstock (TO) location (TO‐1‐30‐07‐2018_COI, TO‐5‐30‐07‐2018_COI, and TO‐6‐30‐07‐2018_COI collected on July 2018). The number of remaining OTUs inferred from the data ranged from 36 to 469 (Table 3). Rarefaction curves of total OTU counts versus the number of sequences calculated for each trap reached asymptote in all cases (Figure 2).

**TABLE 3:** The number of reads, BINs, and sequences associated with BIN identification, remaining OTU counts, percentage of filtered reads (% Filtered), and percentage of dereplicated reads (% Dereplicated) per sample for COI

Sample name	Reads	BINs	Sequences	OTU Counts	% Filtered	% Dereplicated
GT‐1‐03‐07‐2018‐COI	148,806	181	125,219	218	2.45	69.19
GT‐2‐03‐07‐2018_COI	152,732	217	124,416	163	2.43	70.22
GT‐3‐03‐07‐2018_COI	135,306	187	89,030	165	1.18	69.2
GT‐4‐03‐07‐2018_COI	137,956	177	115,974	117	0.7	67.74
GT‐5‐03‐07‐2018_COI	135,926	203	99,710	145	1.77	66.23
GT‐6‐03‐07‐2018_COI	148,600	195	112,480	163	4.05	68.96
WP‐1‐18‐7‐18_COI	160,544	83	5,132	36	60.43	78.78
WP‐2‐18‐7‐18_COI	170,393	159	115,071	117	1.71	70.7
WP‐3‐18‐7‐18_COI	171,886	205	109,977	188	4	67.16
WP‐4‐18‐7‐18_COI	144,228	190	90,246	105	2.3	66.64
WP‐5‐18‐7‐18_COI	141,555	104	85,013	37	11.43	79.07
WP‐6‐18‐7‐18_COI	156,955	172	96,160	60	8.5	69.37
BA‐1_19‐7‐18_COI	188,612	123	129,004	233	8.24	70.17
BA‐2_19‐7‐18_COI	200,026	125	142,661	300	13.69	69.76
BA‐3_19‐7‐18_COI	189,626	191	112,161	259	18.34	67.4
BA‐4_19‐7‐18_COI	200,985	166	134,391	194	13.02	72.98
BA‐5_19‐7‐18_COI	308,781	116	237,742	160	9.48	78.77
BA‐6_19‐7‐18_COI	338,642	189	190,311	410	25.73	70.82
TO‐1‐30‐07‐2018_COI	149,318	217	106,971	254	3.84	67.71
TO‐2‐30‐07‐2018_COI	133,566	195	76,130	358	8.76	66.99
TO‐3‐30‐07‐2018_COI	139,968	128	83,041	320	5.04	70.31
TO‐4‐30‐07‐2018_COI	153,723	135	102,129	243	3.82	69.19
TO‐5‐30‐07‐2018_COI	138,016	284	112,626	224	3.4	67.78
TO‐6‐30‐07‐2018_COI	154,558	313	107,918	316	10.94	66.3

Number of reads, BINs, sequences (Seqs) associated with BIN identification, remaining OTU counts, percentage of filtered reads (Filtered %), and percentage of dereplicated reads (Dereplicated %) per sample for COI						
Sample name	Reads	BINs	Seqs	OTU Counts	Filtered %	Dereplicated %
GT‐1_16‐8‐2018_COI	172,717	194	140,527	158	6.11	68.83
GT‐2_16‐8‐2018_COI	212,186	213	165,688	203	5.27	68.54
GT‐3_16‐8‐2018_COI	254,985	188	204,726	194	3.58	69.25
GT‐4_16‐8‐2018_COI	253,413	217	212,061	135	2.7	69.08
GT‐5_16‐8‐2018_COI	200,655	134	144,174	98	15.66	72
GT‐6_16‐8‐2018_COI	142,747	142	99,940	162	14.57	68.85
WP‐1_22‐8‐18_COI	263,437	157	143,486	347	4.13	69.94
WP‐2_22‐8‐18_COI	330,301	160	225,989	321	0.45	68.26
WP‐3_22‐8‐18_COI_	276,045	205	103,801	452	3.7	66.22
WP‐4_22‐8‐18_COI	267,173	139	177,975	393	3.37	69.25
WP‐5_22‐8‐18_COI	256,504	158	191,390	239	1.34	71.55
WP‐6_22‐8‐18_COI	243,309	121	123,239	82	3.56	76.1
BA‐1_24‐8‐18_COI	145,523	72	62,197	387	14.63	66.75
BA‐2_24‐8‐18_COI	212,628	88	151,481	313	7.74	71.34
BA‐3_24‐8‐18_COI	115,902	63	13,586	469	23.08	62.55
BA‐4_24‐8‐18_COI	102,571	89	69,278	277	7.83	69.16
BA‐5_24‐8‐18_COI	188,706	89	129,043	312	8.02	69.69
BA‐6_24‐8‐18_COI	133,081	60	58,736	339	12.07	67.85
TO‐1_28‐8‐2018_COI	175,589	105	139,710	179	1.7	71.14
TO‐2_28‐8‐2018_COI	167,549	112	100,841	271	9.11	68.75
TO‐3_28‐8‐2018_COI	189,358	103	120,406	356	1.51	68.69
TO‐4_28‐8‐2018_COI	167,074	106	145,180	160	1.44	69.33
TO‐5_28‐8‐2018_COI	231,638	112	189,424	224	4.32	72.79
TO‐6_28‐8‐2018_COI	230,869	112	181,460	102	10.65	71.69

Overall, 2,535 BINs were identified across the 48 traps, representing 57 Orders and 304 Families, the vast majority of them being arthropods (Appendix S1). A total of 5,997,851 sequences were associated with a BIN and together represented an average quality value (QV) score of over 37 out of a total possible score of 40.

### 3.2 Comparative BIN analysis

#### 3.2.1 Traps collected in July 2018

**FIGURE 3:**
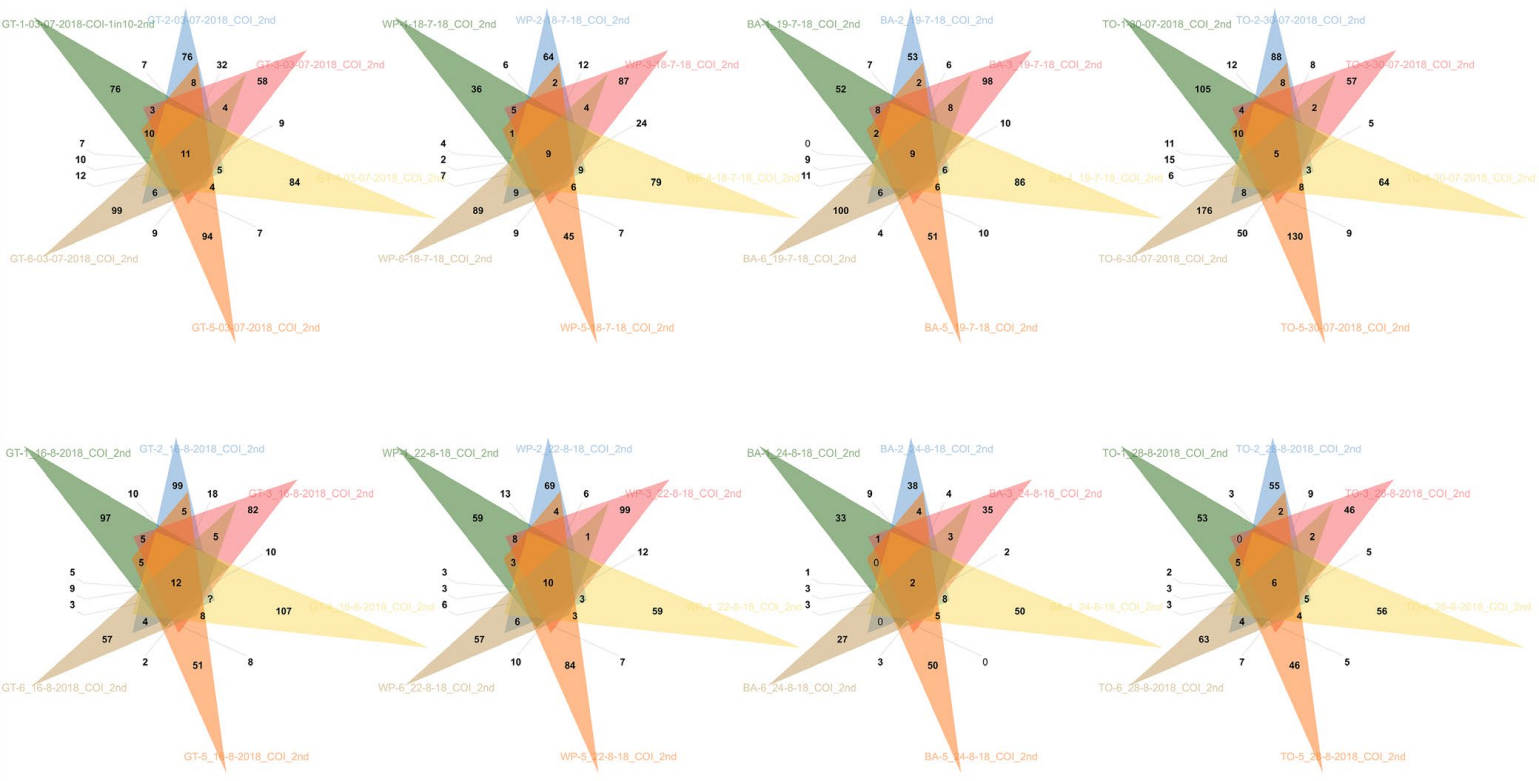
Number of BINs identified per trap and overlapping BINs. Each colored triangle represents one of six traps per location (Halton Hills [GT], Chatham‐Kent [WP], Barrie [BA], and Woodstock [TO]) and date sampled in July and August 2018. The number of overlapping BINs among traps per location and date is indicated in the intercepting area for each representation

A total of 727 BINs were identified across traps placed at the Halton Hills (GT) location, representing 27 Orders and 139 Families. Eleven out of the 727 BINs were shared among the six traps (Figure 3). At the Chatham‐Kent (WP) location, a total of 583 BINs were identified for a total of 29 Orders and 132 Families. Nine out of the 583 BINs were shared among the six traps (Figure 3). At the Barrie (BA) location, a total number of 604 BINs were recognized and distributed among 30 Orders and 140 Families, with nine BINs shared among traps. At Woodstock (TO), 870 BINs were identified; five of them were common among the six traps (Figure 3). Among the 870 BINs, a total of 43 Orders and 183 Families were recovered. Overall, 1,876 BINs were classified in traps placed on southern Ontario, Canada, sites in July 2018. The BINs were distributed among 49 Orders and 261 Families.

#### 3.2.2 Traps collected in August 2018

A total of 701 BINs were identified across traps placed at the Halton Hills (GT) location, with 12 of them shared among traps. Overall, they represented 33 Orders and 141 Families (Figure 3). At Chatham‐Kent (WP), ten BINs were common among traps, with a total of 600 BINs identified and distributed across 34 Orders and 149 Families (Figure 3). A total of 314 BINs were recognized at the Barrie (BA) location, with two of them shared among traps. The 314 BINs were distributed among 26 Orders and 85 Families (Figure 3). At Woodstock (TO), 432 BINs were identified, representing 32 Orders and 112 Families. Six out of the 432 BINs were common among the traps (Figure 3).

In general, for the samples collected in August 2018, a total of 1,360 BINs were identified, and all of them were distributed among 45 Orders and 218 Families.

### 3.3 Identification of plant pests and IAS

#### 3.3.1 Regulated insects

Species‐specific sequence identification was completed with over 97% average mean similarity and over a minimum COI sequence length of 392 bp (Table 4). DNA sequences of two invasive plant pest species regulated by the CFIA (*Lymantria dispar* [gypsy moth] and *A. planipennis* [emerald ash borer beetle]) were detected at the sampling locations, which is not unexpected as the species are known to occur at these locations. DNA sequences of the gypsy moth were also recovered at every sampling location in both collection months. On the contrary, DNA sequences of the emerald ash borer beetle were recovered at only two sampling locations in July (Barrie [BA] and Woodstock [TO]), as well as in August (Halton Hills [GT] and Barrie [BA]) (Table 4). The highest number of gypsy moth sequences was recovered in August at the Halton Hills location. For the emerald ash borer beetle, the highest number of DNA sequences was obtained at the Barrie location in July 2018.

**TABLE 4:** Taxonomy of IAS that impact plant health identified per location.

BIN.Taxon.ID	Phylum	Class	Order	Family	Genus	Species	Seq	% MS	Length
GT 3‐7‐2018									
BOLD:AAA2052	Arthropoda	Insecta	Lepidoptera	Erebidae	*Lymantria*	*Lymantria dispar*	14,434	99.78	396.04
TAX:177812	Arthropoda	Insecta	Lepidoptera	Erebidae	*Lymantria*	*Lymantria dispar*	1	98.39	395
WP 18‐7‐2018									
BOLD:AAA2052	Arthropoda	Insecta	Lepidoptera	Erebidae	*Lymantria*	*Lymantria dispar*	54	99.34	395.19
TAX:24220	Arthropoda	Insecta	Lepidoptera	Erebidae	*Lymantria*	*Lymantria dispar*	1	98.73	395
BA 19‐7‐2018									
BOLD:AAE6821	Arthropoda	Insecta	Coleoptera	Buprestidae	*Agrilus*	*Agrilus planipennis*	214	99.55	394.94
TAX:27304	Arthropoda	Insecta	Coleoptera	Buprestidae	*Agrilus*	*Agrilus planipennis*	4,234	99.73	394.93
BOLD:AAA2052	Arthropoda	Insecta	Lepidoptera	Erebidae	*Lymantria*	*Lymantria dispar*	1	98.48	395
TO 30‐7‐2018									
TAX:27304	Arthropoda	Insecta	Coleoptera	Buprestidae	*Agrilus*	*Agrilus planipennis*	19	99.92	394.89
BOLD:AAE6821	Arthropoda	Insecta	Coleoptera	Buprestidae	*Agrilus*	*Agrilus planipennis*	1	98.73	395
BOLD:AAA2052	Arthropoda	Insecta	Lepidoptera	Erebidae	*Lymantria*	*Lymantria dispar*	138	99.52	398.38
GT 16‐8‐2018									
TAX:27304	Arthropoda	Insecta	Coleoptera	Buprestidae	*Agrilus*	*Agrilus planipennis*	1	97.46	392
BOLD:AAA2052	Arthropoda	Insecta	Lepidoptera	Erebidae	*Lymantria*	*Lymantria dispar*	29,252	99.67	395.04
WP 22‐8‐2018									
BOLD:AAA2052	Arthropoda	Insecta	Lepidoptera	Erebidae	*Lymantria*	*Lymantria dispar*	13	99.93	395
BA 24‐8‐2018									
TAX:27304	Arthropoda	Insecta	Coleoptera	Buprestidae	*Agrilus*	*Agrilus planipennis*	1	99.49	395
BOLD:AAA2052	Arthropoda	Insecta	Lepidoptera	Erebidae	*Lymantria*	*Lymantria dispar*	2	99.50	395
TO 28‐8‐2018									
BOLD:AAA2052	Arthropoda	Insecta	Lepidoptera	Erebidae	*Lymantria*	*Lymantria dispar*	2	99.37	395.5

Note

Included are the reference library's BIN taxon identifier (BIN.Taxon.ID), number of corresponding DNA sequences (Seq), average percent mean similarity (% MS), and average bp length analyzed (Length).

#### 3.3.2 Bacteria

**FIGURE 4:**
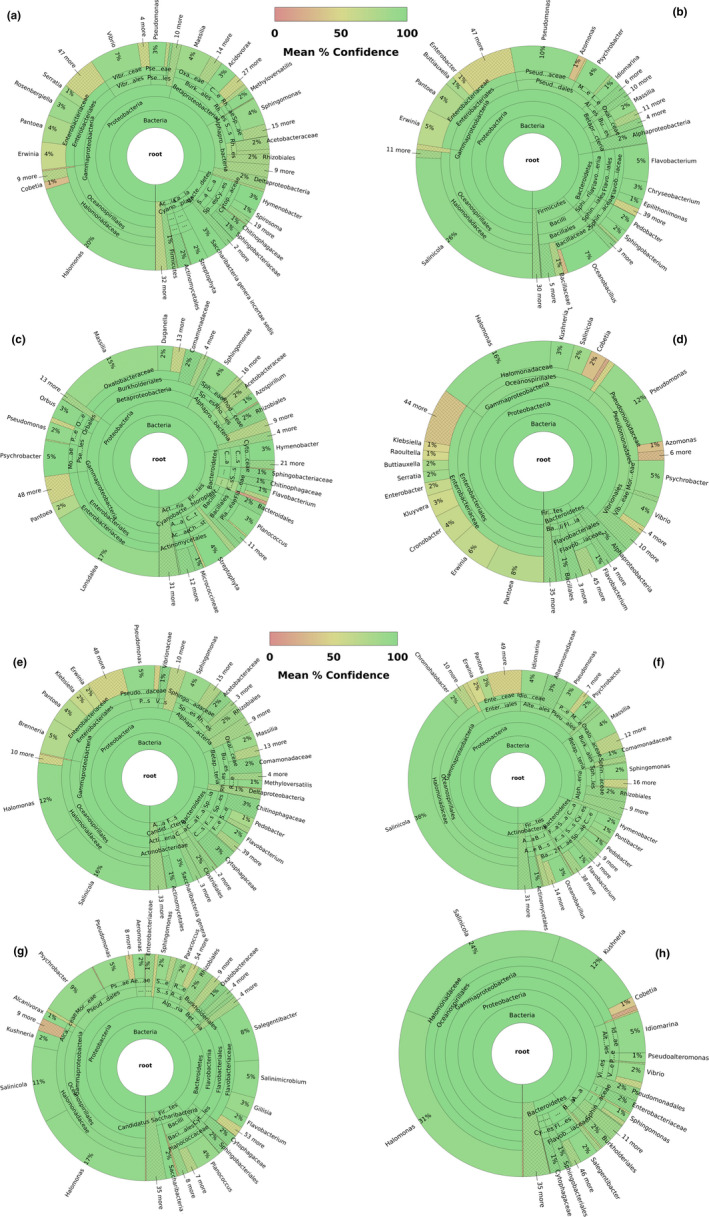
Graph representing bacterial diversity per sampling location and collection date. The color gradient represents mean confidence, as indicated in the gradient bar. An interactive pie chart showing the classification of the sequences and including detailed information for each section of the pie can be viewed online. The list of links per collection sites is indicated below. a: GT 3‐7‐2018 (https://16s.geneious.com/16s/results/fa8ab1e3‐4f50‐4985‐ac59‐7302a31b8451.html); b: wp 18‐7‐2018 (https://16s.geneious.com/16s/results/e7d15b36‐86bd‐493f‐8ccf‐36ac439842c3.html#); c: ba 19‐7‐2018 (https://16s.geneious.com/16s/results/57cc0b9e‐f720‐4476‐9afb‐897e7fcb89fd.html); d: to 30‐7‐2018 (https://16s.geneious.com/16s/results/12fee165‐8bf0‐409f‐adb3‐3501ece47a8d.html#); e: gt 16‐8‐2018 (https://16s.geneious.com/16s/results/386dbe57‐590b‐44ef‐89f6‐f068d64dabff.html#); f: wp 22‐8‐2018 (https://16s.geneious.com/16s/results/a2631a48‐9dd8‐4c6a‐9358‐8f357f012baa.html#); g: ba 24‐8‐2018 (https://16s.geneious.com/16s/results/2dd31759‐6c6c‐458a‐bcc7‐0478626d21d9.html#); h: to 28‐8‐2018 (https://16s.geneious.com/16s/results/f30255aa‐9f0c‐4e4d‐95f1‐8d782cef0f6b.html#)

Five (*Brenneria*, *Pseudomonas*, *Xanthomonas, Xylella*, and *Xylophilus*) out of the seven genera containing species regulated by Canada were identified across collection sites in Ontario and comprised 2,061,117 sequences (https://16s.geneious.com/16s/results/c8f7bebb‐1095‐4a1f‐86e5‐0262cd49e7ab.html). Among the five genera, sequence count and mean confidence ranged from 47 to 103,730 and 27.09 and 93.08, respectively (Figure 4). *Pseudomonas* and *Xanthomonas* exhibited the highest mean confidence values for sequence classification, while *Xylella* showed the lowest value. *Pseudomonas* was also the most represented genus in terms of sequence count (Figure 4).

#### 3.3.3 Fungi

**FIGURE 5:**
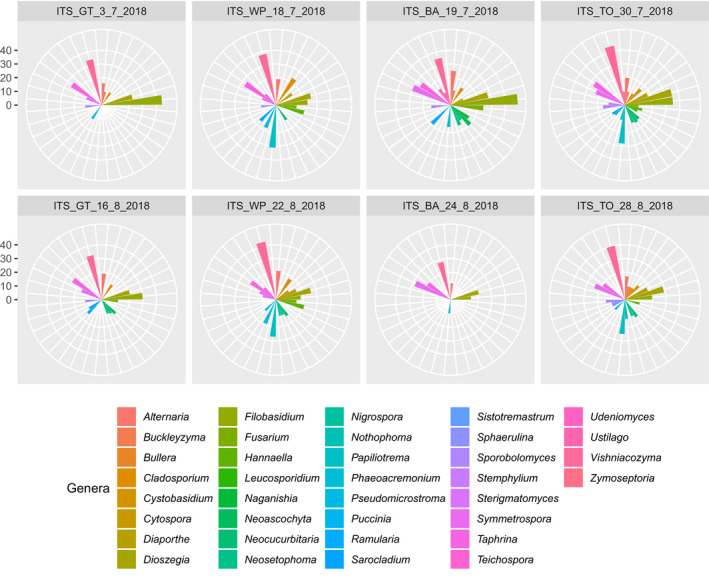
Graphical representation of the fungal genera with ten or more species per sampling location and date. Each genus is represented by a different color, and the scale represents the number of species found

No regulated fungal species were identified across collection sites. A total of 36 genera consisting of ten or more species were detected across sampling locations (Figure 5). Three out of the 36 genera (*Alternaria, Diaporthe,* and *Fusarium*) which have species represented on Canadian regulatory lists were successfully detected with a high percentage of pairwise genetic identity (≥ 98%) in all location sites (Figure 5). The number of fungal genera comprising regulated species per location ranged from three to six, with the traps placed in Barrie in August 2018 containing the lowest fungal diversity (Figure 5).

## 4 DISCUSSION

### 4.1 Performance of optimized protocol

eDNA metabarcoding allows rapid species identification from complex environmental samples, with higher sensitivity, lower environmental disturbance, and shorter processing times than traditional identification methods (Ruppert et al., 2019). These attributes make the integration of eDNA methods into biosurveillance programs for the detecting IAS an ideal choice. The optimized protocol presented here, using a saturated salt trap solution successfully detected IAS and allowed sample processing from eDNA extraction to HTS at a fraction of the cost compared with the Illumina reference protocol (Table 2). Our results suggest the stability of the eDNA in the collection fluid as well as the preservation of arthropod specimens allowing for morphological species identification and validation. Due to the characteristics of our experimental design, such as eDNA potentially remaining in the salt solution for up to 4 weeks, there is an expected level of eDNA degradation. Despite possible degradation, the target fragment lengths (above 400 bp) were effectively amplified for all the molecular markers studied, regardless of the origin of the genetic material (i.e., animal, bacterial, or fungal).

Considering that every step of an adapted eDNA metabarcoding protocol can have a particular impact on species detectability, protocol validation is essential (Ruppert et al., 2019). The asymptotic rarefaction curves presented here may suggest that the read depths of our optimized protocol were sufficient to recover the full OTU diversity present in each trap. Nevertheless, it is important to highlight that the curves reflect OTU diversity based on the specific primer sets used in our study (Table 1). Although we selected primers to maximize OTU representation, due to issues such as “PCR dropout,” primer binding and therefore species detection may have been less than 100% (Griswold, 2019).

Contrary to eDNA metabarcoding of samples collected in ethanol (Zenker et al., 2020), our protocol was successful in generating high‐quality library preparations of the three targeted molecular markers for all of our samples. This validates that salt‐saturated solutions can act as a reservoir for eDNA in conventional trapping surveys (Young et al., 2020). If this were not the case, any logistical advantages over the alcohol‐based fluids listed above would more likely be irrelevant. However, additional studies, specifically addressing eDNA preservation in saturated salt solutions versus alcohol‐based solutions, will be required in samples of known taxonomic composition to compare the methods' efficiency comprehensively.

Our procedure was effective in detecting IAS from high‐risk sites in southern Ontario. Among the 2,535 BINs identified, two regulated insect species known to be established in the area, *A. planipennis* (order Coleoptera) and *L. dispar* (order Lepidoptera), were identified and also validated by a more than 97% mean similarity over a minimum of 392 bp (Table 4). Species were identified using two independent data analysis pipelines to significantly reduce the likelihood of false positives.

Despite the lack of detection of regulated bacterial and fungal species, there were five bacterial (Figure 4) and three fungal genera (Figure 5) comprising regulated species that were successfully recovered across all sampling sites. These results validate our modified protocol for detecting regulated bacterial and fungal taxa based on 16S and ITS and reinforce its utility for the identification of a wide variety of regulated taxa. However, whether or not some taxa are underrepresented in salt trap‐saturated solutions, as has been demonstrated for alcohol‐based collection fluids (Zenker et al., 2020), should be further evaluated.

### 4.2 Applications for biomonitoring and biosurveillance

Since its introduction into North America, the emerald ash borer beetle (order Coleoptera) has been responsible for killing several millions of ash trees across the continent (Herms & McCullough, 2014). Current management plans are mostly focused on biological and insecticidal control of infected areas and do not typically include early detection of the species as it moves into new areas. This approach mainly reflects difficulties in early detection via conventional surveys (Herms & McCullough, 2014). In that respect, present eDNA metabarcoding protocols are promising and allow for not only early detection but also species identifications at any life stage. The containment of pest insect outbreaks is also greatly benefited from the development of nondestructive eDNA metabarcoding protocols that allow early detection. By delineating the boundaries of an outbreak as clearly as possible, actions can be taken on infected areas while avoiding the spread of the pest. The gypsy moth (order Lepidoptera) is listed among the 100 worst IAS by the International Union for Conservation of Nature (IUCN), and it is considered among the worst hardwood defoliators (Vivek et al., 2020). Our sampling protocol was able to trap a regulated Lepidopteran species, even if the specific traps and lures targeted mainly Coleopterans, particularly wood‐boring beetles. This demonstrates the effectiveness of the protocol in detecting IAS and its potential wider applicability beyond our target taxa.

Developing efficient and cost‐effective protocols for the early detection of IAS is key in decreasing tree deaths and related economic losses. In the recent past, the high cost of HTS platforms prohibited their application in routine biodiversity monitoring and biosurveillance programs (Shokralla et al., 2012). Although HTS has become more economically accessible, it constitutes just one element in multistep metabarcoding protocols. In our study, we used alternative cost‐effective chemistries without compromising protocol efficiency and were able to process samples at a reduced cost (Table 2). This cost‐effectiveness if applied to similar programs, could open opportunities for increased replication and thus more accurate species diversity delineations. Although technical replicates were not included in our experimental design, these could be incorporated in the future to evaluate the variability of the testing protocol. Nevertheless, the multiple traps per location could be considered biological replicates when it comes to the evaluation of the presence/absence of IAS in a given location.

Also, as a nondestructive method, our present protocol allows for the morphological identification of the pests in the collection jars. However, even in the absence of morphological confirmation, environmental managers and regulators could direct attention and resources to areas where positive detection of IAS eDNA has been made. In these instances, temporal (multiyear) sampling protocols would be desirable to ensure proper decision‐making and implementation of eradication strategies in infected areas.

It is worth mentioning that morphological identification of arthropods remains a challenge due to factors such as unidentifiable early life stages, specimens escaping from the trap, and advanced or total degradation of specimens in collection jars. In all these cases, the eDNA metabarcoding protocol described here would allow the detection of the species, whereas morphological confirmation may not be possible, leading to false negatives. However, the eDNA of an invasive species that has not been captured can be released with a specimen’s feces and result in potential false‐positive results for a particular location while at the same time signalling the presence of the IAS in the wider region. The eDNA from a non‐captured species can also be found when the invasive species’ material persists in the trapped species’ gut content. These scenarios illustrate how eDNA and morphological identification might yield different species' detection results.

## 5 CONCLUSIONS

Here, we standardized, optimized, and validated a protocol for metabarcoding using eDNA collected from salt trap solutions. We demonstrate the validity of employing eDNA‐based detection of government‐regulated pests as a potential regulatory and management tool. Also, the current protocol supports the utility of salt‐saturated solutions as reservoirs of eDNA and as a valid substitute for alcohol‐based collection fluids in biosurveillance studies.

In an environmental setting, direct applications of the protocol at the national level could include contributing to pest and pathogen databases maintained by the CFIA, the Ministry of Environment and Climate Change Canada, Ministry of Agriculture and Agri‐Food, and Ministry of Health. The protocol may also find utility with similar agencies internationally. Our protocol is not only relevant to CFIA's Plant Health business line but also to their Animal Health business line and to other stakeholders, such as industry, environmental consultants, researchers, and conservation managers. Further, our metabarcoding protocol has potential diagnostic applications in a human health clinical setting for bacterial‐ and fungal‐borne diseases. Importantly, the cost‐effectiveness of the optimized protocol could be useful in both environmental and human health settings where the costs of pathogen detection and identification can be prohibitive. The protocol could be used to investigate and describe pathogen interactions with each other and with their environment (i.e., the pathobiome). Altogether, the validated and cost‐effective protocol may be fundamental in characterizing interactions among humans, animals, plants, microorganisms, and their shared environment to achieve better health results for all (e.g., One Health approach).

## CONFLICT OF INTEREST

None declared.

## AUTHOR CONTRIBUTIONS

**Yoamel Milián‐García:** Data curation (lead); formal analysis (lead); investigation (equal); methodology (equal); writing – original draft (lead); writing – review and editing (equal). **Robert Young:** Conceptualization (equal); investigation (equal); methodology (equal); writing – review and editing (equal). **Mary Madden:** Investigation (equal); methodology (equal); writing – review and editing (equal). **Erin Bullas‐Appleton:** Funding acquisition (equal); resources (equal); writing – review and editing (equal). **Robert H. Hanner:** Conceptualization (equal); funding acquisition (lead); investigation (equal); project administration (lead); resources (equal); supervision (lead); writing – review and editing (equal).

## Supporting information

Appendix S1

## Data Availability

Data available from the University of Guelph Research Data Repository https://doi.org/10.5683/SP2/HTSZNS will become available upon acceptance of the manuscript for publication.
